# All is not lost, when lead goes in the wrong direction

**Published:** 2007-08-01

**Authors:** Uma N Srivatsa, Padraig O'Neill

**Affiliations:** UC Davis Medical Center, Sacramento, CA

**Keywords:** Left SVC, defibrillator

## Abstract

Left sided superior vena cava (SVC) is an uncommon anomaly noted in the general population. It adds complexity to the procedure, when attempting to place pacing or defibrillator devices into the heart. Here we report a case where the leads were placed through the left sided SVC into the right sided chambers giving an interesting X-ray appearance.

Seventy two year old female with dilated cardiomyopathy and left bundle branch block, was referred for cardiac resynchronization therapy. She previously had single chamber defibrillator and had developed worsening heart failure over the years. Under fluoroscopy, her  defibrillator lead was noted to be entering the heart from the left side. With contrast injection from left antecubital vein, she was found to have persistent left superior vena cava ( SVC) and absent innominate vein. Dye flow was extremely rapid from left SVC through coronary sinus (CS), that it was difficult to visualize the branches of coronary sinus. When contrast was administered in the coronary sinus, after occluding SVC and CS os, no significant branches were noted to be present. Therefore LV lead could not be placed by endocardial approach. However atrial lead was placed through the CS into the right atrium, giving this unusual but interesting appearance of the chest X-Ray. (Figure 1,Figure 2). She subsequently received epicardial lead to the left ventricle by mini thoracotomy.

The incidence of Left SVC is about 0.3% of the general population as evident  from autopsy studies, and about 0.47% of  patients receiving pacemaker and defibrillator devices [1]. In a case series among  the158 patients receiving biventricular pacing devices, 4 were found to have left sided SVC, but the patients had tributaries  enabling endocardial placement of the CS lead [2]. Since, left sided placement of the defibrillator can has lower defibrillation energy requirement, it presents a technical challenge, sometimes making it necessary to place the lead from the right side, then tunnel it to the left side where the defibrillator can is placed [3].

## Figures and Tables

**Figure 1:**
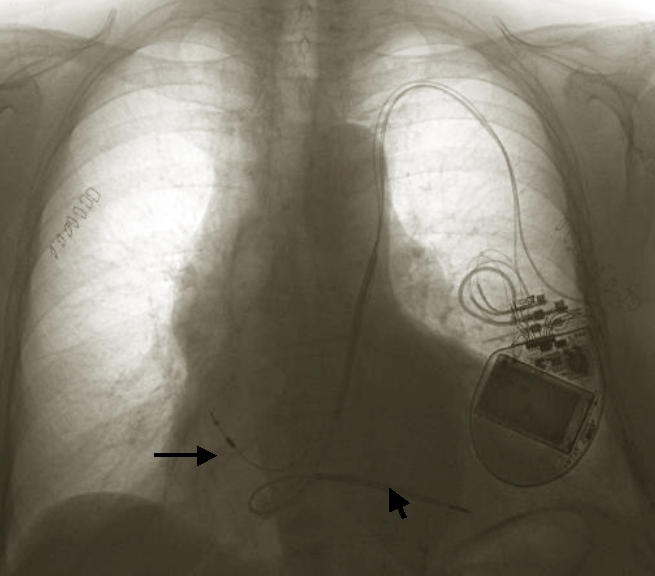
CXR PA view reveals defibrillator on the left chest, RV defibrillator lead (short arrow), RA lead (long arrow) placed through the Left SVC and coronary sinus into the corresponding chambers.

**Figure 2:**
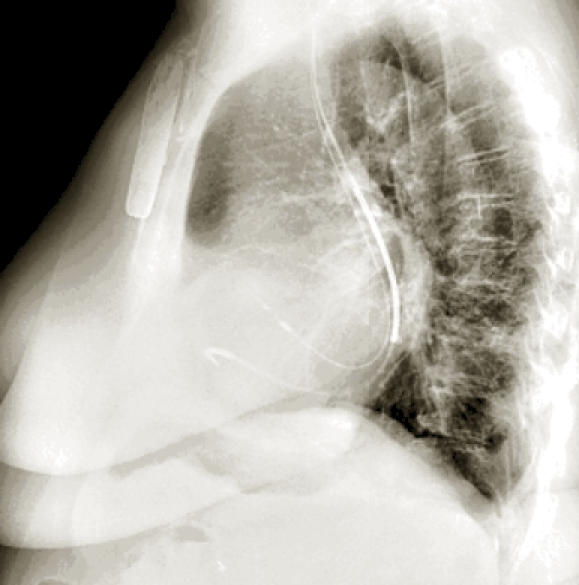
Lateral view: RA and RV leads seen entering from posterior aspect to RA and RV.
